# Link Brain-Wide Projectome to Neuronal Dynamics in the Mouse Brain

**DOI:** 10.1007/s12264-024-01232-z

**Published:** 2024-05-31

**Authors:** Xiang Li, Yun Du, Jiang-Feng Huang, Wen-Wei Li, Wei Song, Ruo-Nan Fan, Hua Zhou, Tao Jiang, Chang-Geng Lu, Zhuang Guan, Xiao-Fei Wang, Hui Gong, Xiang-Ning Li, Anan Li, Ling Fu, Yan-Gang Sun

**Affiliations:** 1 Britton Chance Center for Biomedical Photonics, Wuhan National Laboratory for Optoelectronics, Huazhong University of Science and Technology, Wuhan, 430074 Hubei China; 2 MoE Key Laboratory for Biomedical Photonics, Huazhong University of Science and Technology, Wuhan, 430074 Hubei China; 3 Advanced Biomedical Imaging Facility, Huazhong University of Science and Technology, Wuhan, 430074 Hubei China; 4 Institute of Neuroscience, Key Laboratory of Brain Coginition and Brain-inspired Intelligence Technology, Center for Excellence in Brain Science and Intelligence Technology, Chinese Academy of Sciences, Shanghai, 200031 China; 5 University of Chinese Academy of Sciences, Beijing, 100049 China; 6 School of Future Technology, University of Chinese Academy of Sciences, Beijing, 100049 China; 7 HUST-Suzhou Institute for Brainsmatics, JITRI Institute for Brainsmatics, Suzhou, 215123 China; 8 School of Biomedical Engineering, Hainan University, Haikou, 570228 China; 9 School of Physics and Optoelectronics Engineering, Hainan University, Haikou, 570228 Hainan China

**Keywords:** *In vivo* imaging, fMOST, AO imaging, Deep brain regions

## Abstract

Knowledge about the neuronal dynamics and the projectome are both essential for understanding how the neuronal network functions in concert. However, it remains challenging to obtain the neural activity and the brain-wide projectome for the same neurons, especially for neurons in subcortical brain regions. Here, by combining *in vivo* microscopy and high-definition fluorescence micro-optical sectioning tomography, we have developed strategies for mapping the brain-wide projectome of functionally relevant neurons in the somatosensory cortex, the dorsal hippocampus, and the substantia nigra pars compacta. More importantly, we also developed a strategy to achieve acquiring the neural dynamic and brain-wide projectome of the molecularly defined neuronal subtype. The strategies developed in this study solved the essential problem of linking brain-wide projectome to neuronal dynamics for neurons in subcortical structures and provided valuable approaches for understanding how the brain is functionally organized via intricate connectivity patterns.

**Supplementary Information:**

The online version contains supplementary material available at 10.1007/s12264-024-01232-z.

## Introduction

The brain is composed of diverse subtypes of neurons that form complex neural networks by local and long-range synaptic connections. Both connectivity patterns and neural dynamics are critical for the functions of neuronal ensembles. The development of technology in mesoscopic connectome [1–4] and functional imaging with cellular resolution [5, 6] has made it possible to reveal how neurons in diverse brain regions are structurally organized or functionally concerted. However, in most cases these two sets of information were collected separately, making it difficult to understand how neurons with specific neuronal dynamics convey the information to downstream brain areas.

Several recent studies have developed strategies aimed at solving this problem. In a recent study, a method named 2-SPARSE combined *in vivo* two-photon imaging and plasmid electroporation to label target neurons with distinct calcium dynamics and collected the morphological data by whole-brain imaging with fluorescence micro-optical sectioning tomography (fMOST) [7]. In another study, the same group of neurons was sparsely labeled with GCaMP6m for measuring the neural dynamics and mapping the projectome of neurons in the visual cortex via two-photon imaging and high-definition fluorescence micro-optical sectioning tomography (HD-fMOST), respectively [8]. These methods are not directly applicable to deep brain areas, despite that these two approaches could be applied to map the projectome of functionally defined neurons in the cortex.

Gradient refractive index (GRIN) lenses have been widely used as optical tools for accessing deep brain regions, enabling the acquisition of calcium signals through combinations with one-photon or two-photon microscopy [9–13]. However, the inherent aberrations of a GRIN lens greatly distort neuronal morphology and reduce the effective field of view (FOV) [14–16], thereby impeding the registration between *in vivo* imaging and *in vitro* morphological imaging. In addition, neural networks are composed of diverse types of neurons with different transcriptomic properties. Therefore, it is critical to explore the functional and structural properties of molecularly defined neurons. However, it has been difficult to map the neural dynamics and brain-wide projectome for the same neuron of molecularly defined neuronal subtypes, as the technology to obtain functional and structural data for the same molecularly defined neurons was not available.

In this study, we developed strategies to map the projectome of functionally relevant neurons in brain regions at multiple depths of head-fixed wake mice, including the somatosensory cortex, dorsal hippocampus, and substantia nigra pars compacta. Moreover, we have also achieved the specific sparse labeling of dopaminergic neurons, and determined neural dynamics and brain-wide projectome for dopaminergic neurons in the substantia nigra pars compacta. The same strategy could be applied to map the brain-wide projectome of functionally defined neurons with distinct molecular signatures.

## Materials and Methods

### Animals

Male C57BL/6J and DAT-Cre mice at the age of 6–13 weeks were used for experiments. Mice were injected with the virus at 6 weeks. After viral expression for 6-7 weeks, mice were sacrificed for fMOST imaging. C57BL/6J mice were purchased from the SLAC laboratory (Shanghai). DAT-Cre (JAX006660) mice were initially acquired from Jackson's laboratory. All mice were raised on a 12-hour light/dark cycle (light on at 7:00 am) with ad libitum food and water. All procedures were approved by the Animal Care and Use Committee of the Center for Excellence in Brain Science & Intelligence Technology, Chinese Academy of Sciences, Shanghai, China.

### Virus

The AAV2/8-CaMKII-Cre (1 × 10^13^ v.g./mL) was purchased from WZ Biosciences Inc., (Shandong, China). The AAV2/9-hEF1a-DIO-EYFP-WPRE-pA (1 × 10^13^ v.g./mL), AAV2/8-hSyn-Cre-WPRE-pA (1 × 10^13^ v.g./mL), AAV2/5-hEF1a-DIO-Flpo-WPRE-pA (1 × 10^13^ v.g./mL), AAV2/5-CAG-fDIO-jGCaMP7b-WPRE-pA (1 × 10^13^ v.g./mL) and AAV2/9-hSyn-FLEX-GCaMP6s-WPRE (2 × 10^13^ v.g./mL) were purchased from Shanghai Taitool Bioscience Co. Ltd., (Shanghai, China). pGP-AAV-hSyn-FLEX-jGCaMP7b-WPRE was a gift from Douglas Kim & GENIE Project (Addgene plasmid # 104493; http://n2t.net/addgene:104493; RRID:Addgene_104493) [17] and packaged into AAV2/9-hSyn-FLEX-jGCaMP7b-WPRE (2 × 10^13^ v.g./mL) by the gene editing platform of the Center for Excellence in Brain Science & Intelligence Technology, Chinese Academy of Sciences, Shanghai, China.

### Surgeries

Mice were anesthetized with a mixture of xylazine hydrochloride and zeolite (a mixture of tiletamine hydrochloride and zolazepam hydrochloride) for all surgeries and stabilized on a standard stereotaxic frame (RWD Instruments, China). Ophthalmic ointment was applied to maintain eye lubrication. All stereotaxic injections were performed with a hydraulic manipulator (Narashige, MO10). Mice were allowed to recover from anesthesia on a heating blanket before returning to the home cages.

### S1BF Surgery

Wild-type C57BL/6J mice were used for viral injection in the S1 study. To map the projectome of functionally defined neurons in S1BF L2/3, AAV2/8-CaMKII-Cre (diluted to 6 × 10^9^ v.g./mL) and AAV2/9-hSyn-FLEX- jGCaMP7b-WPRE (2 × 10^13^ v.g./mL) was mixed at the ratio of 1:1. Window implantation was performed as previously described [18]. Briefly, a circular craniotomy (~2.5 mm in diameter) was performed over the left S1BF (coordinate: AP 1.8 mm; ML 2.8 mm), leaving the dura intact. The viral mixture of AAV2/8-CaMKII-Cre and AAV2/9-hSyn-FLEX-jGCaMP7b-WPRE was slowly injected in 2 sites of S1BF (interspaced by 0.4 mm, 20 nL for each injection site, ~0.3 mm beneath the cortical surface). The injection pipette was withdrawn 5 min after the viral injection. An imaging window constructed with a metal cannula and a glass coverslip (2.5 mm in diameter, 0.15 mm in thickness) was implanted over S1, and fixed with dental cement. A titanium post was implanted on the skull for head fixation using 3M Vetbond and dental cement.

To establish the mesoscopic projectome of neurons in S1BF L2/3, a binary AAV expression system was used to sparsely label these neurons. Briefly, AAV2/8-CaMKII-Cre was diluted with 0.01 mol/L phosphate-buffered saline (PBS) to 2 × 10^9^ v.g./mL, and mixed with AAV2/9-hEF1a-DIO-EYFP-WPRE-pA at the ratio of 1:1. Viral mixture was injected in 4 sites in the left S1BF and 1–2 sites in the right S1BF (interspaced by 0.2-0.4 mm, 20 nL for each injection site).

### dCA1 Surgery

To map the projectome of functionally defined neurons in the dCA1, AAV2/8-hSyn-Cre-WPRE (diluted to 3–9 × 10^8^ v.g./mL) and AAV2/9-syn-FLEX- jGCaMP7b-WPRE or AAV2/9-hSyn-FLEX-GCaMP6s-WPRE (2 × 10^13^ v.g./mL) were mixed at the ratio of 1:1. Wild type C57BL/6J mice were injected with 20–40 nl viral mixture in the right dCA1 (coordinate: AP -2.06 mm, ML 1.35mm, DV -1.3mm relative to bregma). The injection pipette was withdrawn 10 min after the viral injection. One week after viral injection, a GRIN lens (1.0 × 8.2 mm, NEM-100-40-10-520-DS, GRINTECH GmbH) was implanted above the dCA1 as previously reported [19]. Briefly, after disinfection with medical alcohol and exposure of the skull, a 1.2 mm-diameter craniotomy was performed over the dCA1 (the center coordinates the same as the virus injection). To prevent the compression of the cortical tissue above dCA1, a cylindrical column of cortical tissue was removed by aspiration using a 26-gauge blunt needle connected to a vacuum pump (LONGER, China). As approaching the corpus callosum or the hippocampus, a 28-gauge or 30-gauge needle was used respectively. During the tissue aspiration procedure, exposed tissue was continually irrigated with a sterile saline solution. After the bleeding stopped, a GRIN lens was carefully and slowly implanted to ~200 μm above dCA1. The GRIN lens was secured on the skull with UV-curable adhesive (CharmFil plus, Korea) and dental cement (Super-Bond C&B). Then a metal head plate was mounted on the skull for head fixation using dental cement.

### SNc Surgery

To map the projectome of functionally defined DA neurons in SNc, AAV2/5-hEF1a-DIO-Flpo-WPRE (diluted to 2.4 × 10^9^ v.g./mL) and AAV2/5-CAG-fDIO-jGCaMP7b-WPRE (1.7 × 10^13^ v.g./mL) were mixed at the ratio of 1:1. DAT-Cre mice were injected with 50 nL viral mixture of AAV2/5-hEF1a-DIO-Flpo-WPRE and AAV2/5-CAG-fDIO-jGCaMP7b-WPRE in the right SNc (coordinate: AP -3.07 mm, ML -1.45 mm, DV -4.2 mm relative to bregma). The injection pipette was withdrawn 10 min after the viral injection. Two weeks after viral injection, a GRIN lens (0.50 × 8.00 mm, CLHS050GFT009, GoFoton) was implanted above the SNc. Briefly, after disinfection with medical alcohol and exposure of the skull, a 0.6-mm-diameter craniotomy was performed over the SNc (the center coordinates the same as the virus injection). To facilitate the implantation of the GRIN lens, a wedge-shaped optic fiber (0.4 mm in diameter) was slowly inserted into the area ~200 μm above the target SNc location and held in position for at least 10 min. After withdrawing the wedge-shaped optic fiber, the lens was implanted to ~100 μm above SNc. The GRIN lens was secured on the skull with UV-curable adhesive (CharmFil plus, Korea) and dental cement (Super-Bond C&B). Then a metal head plate was mounted on the skull for head fixation using dental cement.

### Whiskers Stimulation

Contralateral whiskers were stimulated by air puff or moveable wall pushed by a stepper motor. Air was delivered through a rubber hose connected to a nitrogen cylinder. The hose tip was toward the whiskers at a distance of ~1 cm. The air puff was delivered by opening an electromagnetic valve controlled by the Arduino board. The air pressure was ~228 kPa, indicated by the cylinder valve. For each simulation, after 2 s as a baseline, air puff was delivered at 2 Hz for 2 s, followed by a 13-s delay to initiate another trial.

The moveable wall (2 cm × 2 cm, 2 mm thick) was made of resin via 3D printing, glued to a mini-stepper motor. The movement of the wall was driven by the stepper motor controlled by the Arduino board. The wall was 0.7 cm away from the tips of the whiskers at the beginning of each trial. After 2 s as a baseline, the wall began to move toward the whiskers, attaching the whiskers in 1 s, and stopped moving toward the face at about 4 mm to the face to make sure the touch stimuli were only applied to the whiskers. The wall stepped back and forward at a short distance (~3 mm) twice, making the whiskers vibrate. Then the wall was retracted to the original location. The duration of touch stimuli was 4 s. There was a 12-s delay before initiating another trial.

There were 15 trials in total for each imaging plane. Trials with and without touch stimuli (air puff or moveable wall) were randomly arranged.

### Foot-Shock Stimulation

Electric shocks (1 s, 0.8 mA) were delivered to the feet of the mouse by a customized shock grid and a shock generator (Anilab Scientific Instruments Co., Ltd., China). There were 20 trials in total for each imaging plane. Each trial consisted of a randomized interval (25–30 s) followed by a 1 s foot-shock. Mice were typically imaged for 3–5 planes.

### *In Vivo* Calcium Imaging

Two-photon imaging was performed in S1BF and dCA1, while confocal imaging was performed in SNc. All imaging was performed 2 or 4 weeks after the cranial window or GRIN lens implantation respectively. Mice were trained to adapt to head fixation for 30 min per day for 7 days before the experiments to reduce the motion artifact.

For the S1BF part, calcium imaging was performed via a custom-built two-photon microscope (https://wiki.janelia.org/wiki/display/shareddesigns/MIMMS), with a resonant galvanometer (Thorlabs; 16 kHz line rate, bidirectional). jGCaMP7b was excited at 920 nm using a Ti-Sapphire laser (Chameleon Ultra II, Coherent). Emitted fluorescence was detected using GaAsP photomultiplier tubes (10770PB-40, Hamamatsu). Images (512 × 512 pixels, ~480 × 430 μm) were acquired through a Nikon 16×, 0.8 NA water-immersion objective at ~30 Hz using ScanImage. Due to the dispersive distribution of labeled neurons in S1BF L2/3, there were multiple imaging planes to include neurons as many as possible. After recording neural responses to touch stimulation and spontaneous activity, z-stack images were acquired to get the 3D information of imaged neurons.

For the dCA1 part, *in vivo* imaging was performed with a commercial multi-photon fluorescence microscope (A1MP, Nikon). jGCaMP7b or GCaMP6s was excited at 920 nm using a Mai Tai HP Deepsee laser. Emitted fluorescence was detected by a GaAsP photomultiplier tube. For dynamics recording, images (512 × 512 pixels, ~1.0 × 1.0 mm) were acquired through a Nikon 10×/0.45 NA air objective at ~15 Hz by a resonant scanner. After dynamics recording, z-stack images were acquired by a commercial multi-photon fluorescence microscope or custom-built two-photon adaptive-optics microscope to get the 3D information at ~1 Hz by a pair of galvanometers. The adaptive optics two-photon microscopy was built upon a homebuilt two-photon microscope with a modal-based AO method implemented. Briefly, an 800-nm femtosecond laser output (Insight X3, Spectral Physics) was attenuated with a half-wave plate and a polarizing beam splitter cube and then expanded to overfill the display panel of a reflective phase-only liquid crystal SLM (X13138-07, Hamamatsu). The beam was scanned by a pair of galvanometers (6-mm aperture, 6215H, Cambridge Technology). Then the fluorescence generated by two-photon excitation was collected by a photomultiplier tube (H7422-40, Hamamatsu). An air objective (CFI Plan Apochromat Lambda D, NA 0.45, 10×, Nikon) was used to match the NA of the GRIN lens.

For the SNc part, *in vivo* imaging was performed with a commercial multi-photon fluorescence microscope (A1MP, Nikon). jGCaMP7b was excited at 488 nm using an Argon ion laser. Emitted fluorescence was detected by a photomultiplier tube. For neural dynamics recording, images (512 × 512 pixels, ~1.0 × 1.0 mm) were acquired through a Nikon 10×/0.45 NA air objective at ~15 Hz by a resonant scanner.

### AO Imaging

AO imaging was performed by custom-built adaptive optics two-photon microscopy as previously reported [20]. In our home-built adaptive optics two-photon microscopy, a spatial light modulator (SLM) and model-based AO optimization scheme were combined to measure and correct the intrinsic aberration induced by a GRIN lens. For wavefront measurement, 21 Zernike modes were measured independently. The final wavefront was generated by summing the 21 Zernike modes and updated to SLM. For the measurement of each mode, nine amplitude changes were intentionally added. Several images were obtained as a clue to retrieve the aberrated wavefront. Finally, the mean intensity was used as a quality metric and the best amplitude of the Zernike mode was obtained by Gaussian fitting. The wavefront measurement was usually repeated 2–3 times to get the optimal distorted wavefront. Since the aberration characterization at each position of the GRIN lens was different, the field of view was divided into 3 × 3 subregions. The aberrations of 9 subregions were measured and corrected successively. The obtained 9 correction subimages were stitched together by a stitch algorithm.

### Analysis for *In Vivo* Imaging Data

The motion of calcium images was corrected with a cross-correlation-based registration algorithm for the S1BF part and with *Turboreg* (by registering all frames in an imaging session to an average projection of the whole imaging as the reference frame) for the dCA1 and SNc part. Regions of interest (ROIs) corresponding to visually identified somas were manually drawn using custom-written software in MATLAB. The fluorescence of each neuron was extracted as the average of all the pixels within the ROI along the time series.

Neural responses were represented as ∆F/F_0_ and Z-scores. The ∆F/F_0_ was calculated as $(F(t)-{F}_{0})/{F}_{0}$, where $F\left(t\right)$ is the fluorescence signal value at time t, ${F}_{0}$ is the mean fluorescence signal value over the baseline period. The Z-scores were calculated as $(F\left(t\right)-\overline{x })/ \sigma$, where $F\left(t\right)$ is the fluorescence signal value at time t, and $\overline{x }$ and $\sigma$ are the mean and standard deviation (SD) of the fluorescence signal value over the baseline period.

For imaging sessions with touch stimuli applied to whiskers, the baseline was set from -2 s or -3 s to 0 s, where 0 indicated the onset of touch stimuli (air puff or moveable wall) delivery. A neural response in a trial was identified as touch responsive when the mean ∆F/F_0_ during touch stimuli (0–2 s for air puff, 0–4 s for moveable wall) was higher than 1 × SD of the baseline. A touch-responsive neuron was determined when there were 50% of trails responsive to air puff or moveable wall-delivered touch, and the mean ∆F/F_0_ after stimulus onset was 2 × SD higher than the baseline averaged from all trials of the same stimulus.

For imaging sessions with foot-shock, Ca^2+^ fluorescence traces were obtained by calculating the temporal ∆F/F_0_ from -2 s to 10 s, where 0 indicated the onset of foot-shock delivery. The baseline was set from -2 s to 0 s. A neural response in a trial was considered a foot-shock-related response if the mean ∆F/F_0_ during the response window (0–4 s) was higher than 1 × SD (or lower than -1 × SD) of the baseline. If there were 50% of trials related to foot-shock and the average ∆F/F_0_ was 2 × SD higher or -2 × SD lower than the mean baseline averaged from all trials of the same stimulus, the neuron was considered to be excited or inhibited.

### fMOST Imaging, Cell Reconstruction, and Image Registration

All the mouse brain samples were imaged using the HD-fMOST [2] that combined a line-illumination modulation (LiMo) microscope with thin histological sectioning. The embedded brains were imaged in a water bath containing propidium iodide (PI) at a voxel resolution of 0.325 × 0.325 × 1.0 μm^3^. The HD-fMOST system automatically performed the cycles of imaging and sectioning until the brain-wide data acquisition finished. The raw data was saved at 16-bit depth in LZW compression TIFF format.

There were two imaging channels, jGCaMP7b (for axon tracing) and PI (for image registration) in the fMOST raw image data. Data processing and cell reconstruction for S1BF and SNc were accomplished by the FNT as previously described [4], while neurons in dCA1 were reconstructed in GTree software through human-computer interaction [21]. Two skilled annotators independently worked on the reconstruction of each neuron. Another tracer would determine the differences and merge the two tracing results into one. All reconstructed neurons were saved as SWC files.

To facilitate the projectome analysis, reconstructed neurons under each brain sample Framework should be registered to the Allen Mouse Brain Common Coordinate Framework (CCFv3). First, the characteristic brain regions were manually segmented according to the PI channel and CCFv3. Next, the transformation matrix of each brain sample was calculated by affine transformation and nonlinear transformation. Finally, the matrix was applied to those reconstructed neurons in the brain sample to obtain the reconstructed neuron under the CCFv3.

### Cell Registration of *In Vivo* Imaging and fMOST

The cell registration process between two-photon imaging and HD-fMOST followed the methodology described in the previous report [8]. To determine the imaging field for the S1BF region during two-photon imaging, we relied on the distribution of blood vessels as indicated in the HD-fMOST data. However, in deeper brain regions where blood vessels were difficult to observe, we selected the imaging field of HD-fMOST data according to the GRIN lens track. We relied on the distinctive features of neuronal morphology and their relative positions. Additionally, the imaging depth of neurons from *in vivo* imaging aided in the search for corresponding neurons in HD-fMOST data. The specific procedure was as follows:

First, we cropped the marked areas on the HD-fMOST data. Then, a rigid transformation was performed on the cropped three-dimensional HD-fMOST data to align the cutting direction with the general direction of the two-photon imaging plane, followed by projection. Initial orientation calibration was carried out based on the distribution of blood vessels or the GRIN lens track. The pairing of the *in vivo* data was based on neurons with distinctive morphology features. This involved an affine transformation and pairing relationships established based on the positional relationships of the cell bodies (whether there were other interfering neurons nearby) and morphological features. Finally, for neurons in the HD-fMOST data successfully paired with neurons recorded by *in vivo* imaging, an inverse transformation was applied using the recorded transformation matrix to obtain the corresponding neuron coordinates in the three-dimensional whole brain imaging data.

In addition, due to the smaller FOV, fewer recorded neurons compared to all the labeled neurons and less morphological information of recorded neurons in SNc, neurons were manually registered based on the neuronal distribution and morphology by 5 independent persons. Successful registration was confirmed when at least 3 out of 5 individuals agreed on the alignment.

### Analysis for fMOST Data

The projection strength matrix was calculated using ${\text{ln}}(axon length+1)$ for each brain region. The projection probability for each functional cluster was calculated as the number of neurons projecting to target brain regions divided by the total number of neurons in the corresponding functional clusters. Hierarchical clustering was performed using the Euclidean correlation metric and Ward’s linkage based on the projection strength matrix. The threshold was set to 60 for S1BF neurons.

### Immunohistochemistry

To determine the specificity of viral labeling for DA neurons, mice were sacrificed to dissect the brain after viral expression for 7 weeks. Mice were anesthetized with an intraperitoneal injection of a mixture of xylazine hydrochloride and zeolite. The mice were transcardially perfused with 0.1 mol/L phosphate-buffered saline (PBS) followed by 4% paraformaldehyde (PFA). The brains were dissected and post-fixed with 4% PFA overnight and then rinsed five times with PBS. Coronal sections (50 μm) prepared with a Leica VT1200S vibrating microtome were used for immunohistochemistry. Sections were rinsed 3 times with PBS for 10 minutes and then blocked with 5% normal donkey serum in PBST (0.3% Triton X-100) for 1 hour at room temperature. Sections were stained with the Rabbit anti-TH primary antibody (1:250, Abcam, Cat No. ab137869) at 4 °C overnight and then washed three times in PBST for 10 min, followed by incubating with goat anti-rabbit Alexa Fluor 555 secondary antibody (1:200, Abcam, Cat No. ab150078) at room temperature for 2 h. Then sections were mounted onto glass slides with the mounting medium DAPI Fluoromount-G (SouthernBiotech, Cat. No. 0100-20) and coverslips. Sections were imaged with a laser scanning confocal microscope (LSM710) equipped with a 20× air objective (Plan-Apochromat 20×/0.8). Cell counting was manually carried out.

### Quantification and Statistical Analysis

The sample size of each experiment was provided in the Results and figure legends. The data was presented as mean ± SEM. All statistical analyses were performed using GraphPad Prism 8 and MATLAB 2018a. Statistical significance was set at *P* < 0.05. Student's *t*-test was used for determining the difference in morphology of EYFP- and jGCaMP7b-labeled neurons.

### Data and Materials Availability

fMOST data of S1BF can be visualized and downloaded from the website: 10.12412/BSDC.1706148414.20001. All the other relevant data and code for this study can be made available by the Lead Contact upon reasonable request.

## Results

### Link the Brain-Wide Projectome with Neuronal Calcium Dynamics for Individual Neurons in Somatosensory Cortex

**Fig. 1:**
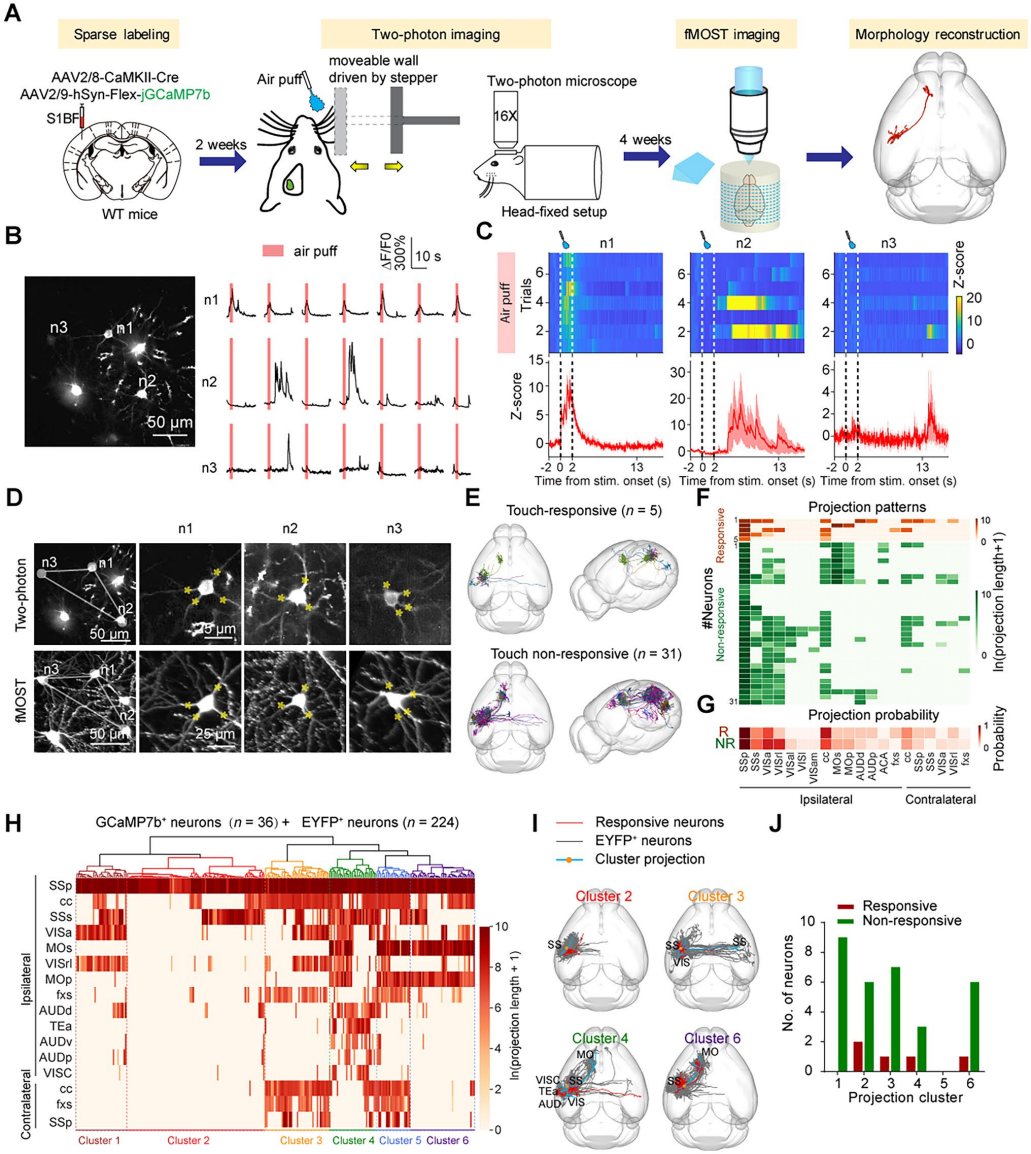
Projectome of functionally defined neurons in S1BF**. A** Schematic diagram illustrating the procedure to map the projectome of functionally defined neurons in L2/3 of S1BF. **B** Neural responses to touch stimuli (Air puff) in an example imaging field. Left, an example field of view. Right, neural responses of 3 neurons indicated in the left in each trial. **C** Heat maps (Top) and averaged trace (Bottom) of the calcium signal of neurons in **B** responding to air puff. The air puff was delivered for 2 seconds as indicated by dashed lines. **D** Cell registration for neurons in **B** between images from two-photon imaging and fMOST. Lines indicate the distribution pattern of neurons, and yellow asterisks identify the same branches.** E** The whole-brain projectome of touch-responsive neurons (Top, *n* = 5) and non-responsive neurons (Bottom, *n* = 31). **F** Projection patterns of individual touch responsive (Top, indicated as R) or non-responsive (Bottom, indicated as NR) neurons in **E**. **G** The probability of touch responsive and non-responsive neurons projecting to the target brain areas. **H** Classification of EYFP-labeled neurons (*n =* 224 neurons from 5 mice) and jGCaMP7b-labeled neurons (*n =* 36 neurons from 2 mice) based on their projection pattern. **I** The whole-brain projectome of 4 projection clusters with touch-responsive neurons. **J** Distribution of touch responsive or non-responsive neurons in 6 projection-defined clusters. SSp, primary somatosensory area; SSs, supplemental somatosensory area; VISa, anterior visual area; VISrl, rostrolateral visual area; VISal, anterolateral visual area; VISl, lateral visual area; VISam, anteromedial visual area; cc, corpus callosum; MOs, secondary motor area; Mop, primary motor area; AUDd, dorsal auditory area; AUDp, primary auditory area; ACA, anterior cingulate area; fxs, fornix system; TEa, temporal association areas; AUDv, ventral auditory area; VISC, visceral area.

The somatosensory cortex is important for processing diverse submodalities of somatosensory information. It has been shown that neurons in the primary somatosensory barrel cortex (S1BF) with different downstream targets exhibited distinct functional dynamics [22]. However, the projectome of functionally defined neurons has not been comprehensively examined for S1BF neurons. Thus, we took S1BF as an example and mapped the brain-wide projectome of S1BF neurons activated by mechanical whisker stimulation via a combination of two-photon calcium imaging and HD-fMOST (Fig. 1A). We sparsely labeled L2/3 neurons in S1BF of mice via injection of AAV-CaMKII-Cre and AAV-hSyn-Flex-jGCaMP7b. To examine touch-responsive neurons, we started to image neuronal dynamics after viral expression for 2 weeks. Due to the large variability of jGCaMP7b^+^ neurons with virus expressing, we continued to sequentially perform two-photon imaging till mice were sacrificed for fMOST imaging. Therefore, two to six weeks after viral injection, we recorded calcium dynamics of L2/3 neurons in S1BF in response to mechanical stimuli applied to whiskers (air puff or moveable wall) via two-photon imaging in awake and head-fixed mice. By analyzing the response pattern of the L2/3 neurons in S1BF to touch stimuli (Fig. 1B, C, and S1), we found that 8.2% of neurons were responsive to touch stimuli (5 out of 61 recorded neurons, Fig. S2). The percentage of touch-responsive neurons was comparable to that reported previously [6]. To examine the projectome of these neurons in L2/3 of S1BF, we acquired the whole-brain image with HD-fMOST at 6 weeks after viral injection (Fig. S3). We registered the neurons imaged with the two-photon imaging to the brain-wide images acquired with HD-fMOST based on their morphology and distribution (Fig. 1D) with a method reported recently [8]. We achieved registration for 43 out of 61 recorded neurons. We chose to reconstruct the morphology of 36 neurons (Fig. S4A-C). These neurons were classified into 2 clusters based on their response property, touch-responsive or non-responsive neurons (Fig. 1E-G). The downstream targets of touch-responsive neurons differed at the single neuron level, although a limited number of touch-responsive neurons. There were also minor differences in the projection probability between touch-responsive and non-responsive neurons, which indicates potential involvement of touch sensory processing, such as secondary somatosensory cortex (SSs).

As S1BF neurons exhibited diverse projection patterns, we determined how many projectome-defined clusters are in L2/3 neurons of S1BF and how touch-responsive neurons are distributed in these clusters. We thus mapped the projectome of these neurons in L2/3 of S1BF by labeling these neurons with EYFP. We found that jGCaMP7b is comparable to EYFP in labeling the neuronal morphology, as no significant differences were found between EYFP and jGCaMP7b labeled neurons in either axon length or the total number of axon branches (Fig. S4). We classified all reconstructed neurons obtained either by jGCaMP7b labeling (*n =* 36) or EYFP labeling (*n =* 224) in L2/3 of S1BF. These neurons could be classified into 6 clusters (clusters 1–6) based on their projection length in target brain areas. For example, cluster 2 neurons primarily projected locally, cluster 3 neurons projected to the contralateral somatosensory cortex, and clusters 5 and 6 neurons projected to the motor cortex. Notably, no touch-responsive neurons were identified in clusters 1 and 5, whereas touch-non-responsive neurons were distributed across five clusters, excluding cluster 5 (Fig. 1I, J). Touch-responsive neurons were distributed in different projection clusters. This result suggests the functionality and structure did not have a simple one-to-one correspondence in the somatosensory cortex, although the number of touch-responsive neurons is limited.

### Link the Brain-Wide Projectome with Neuronal Calcium Dynamics for Neurons in Dorsal CA1

**Fig. 2:**
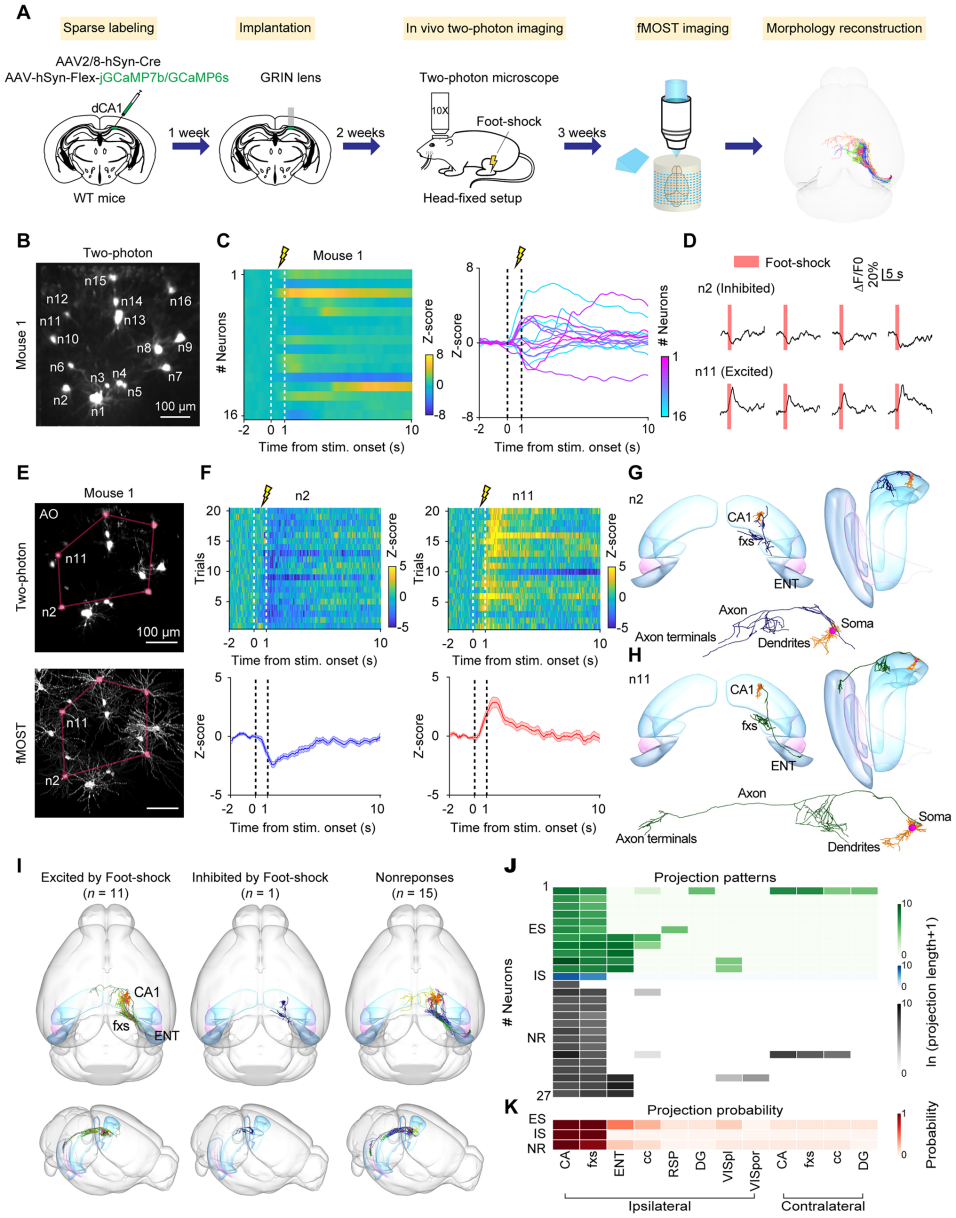
Projectome of neurons in dCA1 with distinct response patterns. **A** Schematic diagram illustrating the procedure to map the projectome of functionally defined neurons in dCA1. **B** Maximum projection of two-photon z-stack imaging without AO. **C** The heatmap (Left) and trace (Right) of averaged neuronal calcium activity of all neurons (Mouse 1) responding to foot-shock. Dashed lines indicate foot-shock delivery. **D** Calcium traces of 2 example neurons from **C** in response to foot-shock in individual trials. The shaded line indicates foot-shock delivery. **E** Cell registration for neurons between two-photon imaging and fMOST data. Top, Maximum projection of two-photon z-stack imaging. Bottom, Maximum projection of fMOST imaging around object side of GRIN lens. Lines indicate the distribution pattern of representative neurons. **F** The heat maps (Top) and averaged trace (Bottom) of the calcium activity of representative neurons indicated in **B, D,** and** E** responding to foot-shock. The dashed line indicates foot-shock delivery. **G** The projectome and entire morphology of a representative IS neuron indicated in **D, E,** and** F**. **H** The projectome and entire morphology of a representative ES neuron indicated in **D, E,** and** F**. **I** The whole-brain projectome of 3 functional clusters of neurons defined by two-photon imaging and fMOST (Excited by foot-shock, ES, *n =*11 neurons. Inhibited by foot-shock, IS, *n =*1 neuron. No response to foot-shock, NR, *n =*15 neurons.). **J** Projection patterns of individual neurons with different response patterns in **I**. **K** The probability of ES, IS and NR neurons projecting to the target downstream regions. CA, Ammon's Horn; fxs, fornix system; ENT, Entorhinal area; cc, corpus callosum; RSP, Retrosplenial area; DG, Dentate gyrus; VISpl, Posterolateral visual area; VISpor, Postrhinal visual area.

Subcortical brain areas play essential roles in diverse physiological functions, such as memory, and emotion [23–26]. However, it remains challenging to simultaneously examine the neuronal dynamics and projectome for neurons in subcortical brain areas at the single-neuron level. Given that the neural activity of hippocampal neurons is highly correlated to their projection targets [27], we thus took the dorsal CA1 hippocampal subregion (dCA1), situated 1 mm below the cortical surface, as an example to develop the strategy for acquiring the neuronal dynamics and brain-wide projectome for the same group of neurons (Fig. 2A). We injected a mixture of AAV-hSyn-Cre and AAV-Flex-jGCaMP7b or AAV-Flex-GCaMP6s virus into the dCA1 region of wild type mice to sparsely label dCA1 neurons. We performed two-photon imaging by integrating a GRIN lens to record neuronal responses to foot-shock in awake and head-fixed mice, which is known to activate dCA1 neurons [28]. We found that the dCA1 neurons exhibited different response patterns to foot-shock (Fig. 2B, C): excited by foot-shock (ES, *n =* 11 neurons), inhibited by foot-shock (IS, *n =* 1 neuron), and non-responsive to shock (NR, *n =* 15 neurons) (Fig. 2C, D and S5).

To facilitate registration between *in vivo* and *in vitro* imaging data, we used a custom-built adaptive optics (AO) microscope to capture a three-dimensional (3D) image of the dCA1 region surrounding the imaging plane [20] (Fig. 2E, Top). This AO microscope effectively corrected the distortions caused by the GRIN lens and improved the neuronal morphology (Fig. S6). Additionally, the fluorescence intensity of neurons and fibers was enhanced (~2.57-fold for neurons and 3.06-fold for fibers, Fig. S6 C-F). We observed that the aberration correction was more effective at the edges of the FOV compared to the center (Fig. S6 B, E, F), which may be due to larger off-axis optical aberrations at the FOV edges [29]. These improvements in neuronal morphology and fluorescence intensity are advantageous for registration between *in vivo* and *in vitro* imaging data.

Next, we obtained a high-resolution whole-brain image dataset using HD-fMOST (Fig. 2E, Bottom and S7). To register the two-photon imaging data to HD-fMOST data, we selected the imaging field of the HD-fMOST data according to the GRIN lens track and projected the selected imaging field along the track to obtain one image of the imaging field. We performed cell registration based on neuronal morphology and distribution and have successfully registered 90 out of 91 neurons in HD-fMOST data (*n =* 3 mice), with one neuron not registered due to the weak brightness. We obtained the brain-wide projectome of functionally relevant neurons (Fig. 2F-H).

Out of the 90 neurons, we were able to reconstruct the morphology of 80 neurons (Fig. S8A). Among these neurons, 27 neurons (33.7%) were fully reconstructed, and 53 neurons (66.3%) were partly reconstructed due to weak fluorescence intensity, axonal winding, or damage by the GRIN lens. Further analysis showed that neurons activated by foot-shock had higher projection probability to the entorhinal area, the posterolateral visual area, and the contralateral hippocampus (Fig. 2I-K). In addition, we found that non-responsive neurons showed similar projection patterns to neurons activated by foot-shock. Thus, these data suggest that dCA1 neurons with similar projection patterns may be heterogeneous in function. Together, by combining deep brain imaging and HD-fMOST, we successfully mapped the brain-wide projectome of dCA1 neurons with distinct response patterns.

### Link the Brain-Wide Projectome to Neuronal Calcium Dynamics of Dopaminergic Neurons in SNc

**Fig. 3:**
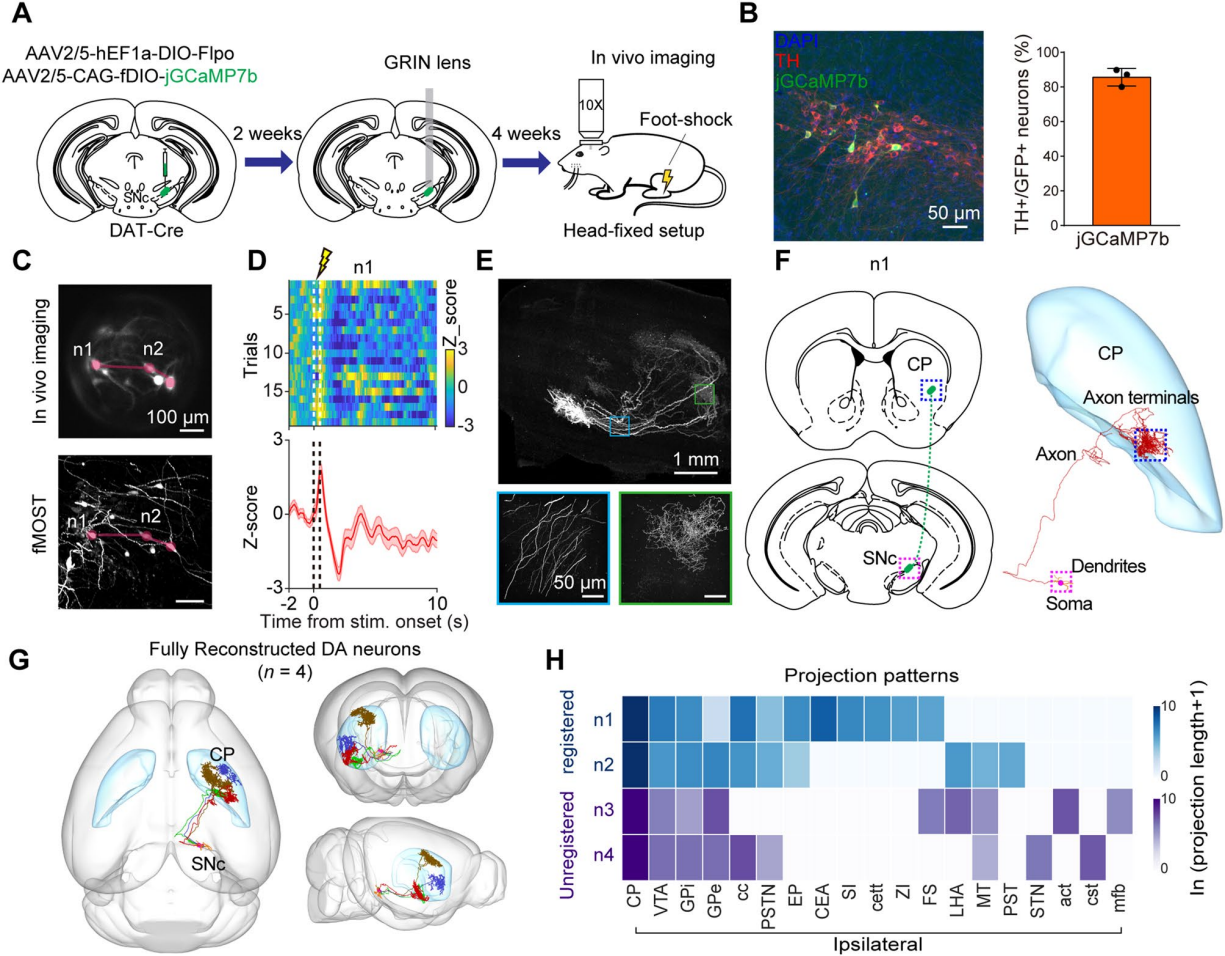
Projectome of DA neurons in SNc responding to foot-shock**. A** Schematic diagram of viral mixture injection and GRIN lens implantation in SNc and *in vivo* imaging in head-fixed mice with foot-shock delivered. **B** An example image showing jGCaMP7b expression (Green) and TH immunostaining (Red) in SNc of DAT-Cre mice (Left). The percentage of TH^+^ neurons in jGCaMP7b^+^ labeling neurons (Right, *n =* 3 mice). **C** Cell registration for neurons between *in vivo* imaging and HD-fMOST imaging. Lines indicate the distribution pattern of representative neurons. **D** The heatmap and trace of representative DA neuron in SNc response to foot-shock. Dashed lines indicate the shock stimuli delivery. **E** Whole-brain projection of sparsely labeled DA neurons in SNc (Top), and enlarged view of boxed regions indicated in the top (Bottom). **F** The schematic diagram (Left) and whole-brain projectome (Right) of a DA neuron indicated in **D**. **G** The whole-brain projectome of fully reconstructed DA neurons (*n* = 4 neurons). **H** The projection patterns of corresponding neurons in** G**. Top, the projection patterns of registered DA neurons. Bottom, the projection patterns of unregistered DA neurons. CP, Caudoputamen; VTA, Ventral tegmental area; GPi, Globus Pallidus internal segment; GPe, Globus Pallidus external segment; cc, corpus callosum; PSTN, Parasubthalamic nucleus; EP, Endopiriform nucleus; CEA, Central amygdalar nucleus; SI, Substantia innominata; cett, cervicothalamic tract; ZI, Zona incerta; FS, Fundus of striatum; LHA, Lateral hypothalamic area; MT, Medial terminal nucleus of the accessory optic tract; PST, Preparasubthalamic nucleus; STN, Subthalamic nucleus; act, anterior commissure, temporal limb; cst, corticospinal tract; mfb, medial forebrain bundle.

The neural network is composed of different subtypes of neurons defined by different molecular signatures, and it is critical to link the brain-wide projectome to neuronal calcium dynamics of molecularly defined neurons. However, the simultaneous study of the neural dynamics and projectome of molecularly defined neurons has not been achieved at the single-neuron level. We thus developed the strategy to link the projectome to neuronal calcium dynamics for molecule-defined neuronal subtypes. Here, we took dopaminergic neurons in the substantia nigra pars compacta (SNc) as an example. The key issue is to achieve sparse labeling of dopaminergic neurons with jGCaMP7b. We injected a mixture of AAV-hEF1a-DIO-Flpo and AAV-CAG-fDIO-jGCaMP7b viruses into the SNc region of DAT-Cre mice to sparsely and specifically label the DA neurons (Fig. 3A). We labeled ~7 neurons in a 708 × 708 μm FOV (Fig. 3B, Left), indicating the sparsity of this labeling strategy. We found that more than 86.1% of the jGCaMP7b-labeled neurons were positive for tyrosine hydroxylase (99 TH^+^ neurons out of 115 GFP^+^ neurons, *n =* 3 mice), a marker for dopaminergic neurons (Fig. 3B, Right), suggesting the specificity of this labeling strategy. We implanted a GRIN lens above the injection site for subsequent *in vivo* calcium imaging in awake and head-fixed mice.

However, the fluorescent brightness of the binary recombinases-dependent strategy was weaker than that of the strategy used in dCA1 (Fig. S9). In addition, the fluorescence signal of two-photon excitation is weaker by several orders of magnitude compared to the one-photon [30]. We chose to use confocal imaging to record the neural dynamics in SNc. To avoid the photobleaching and phototoxicity caused by long-term imaging, we imaged the neuronal responses to foot-shock in a trial-by-trial manner, which is known to activate dopaminergic neurons [31, 32]. We obtained 7 dopaminergic neurons (Fig. 3C, Top) during *in vivo* calcium imaging, and these neurons exhibited different response patterns to the foot-shock (Fig. 3D and S10A). To map the brain-wide projectome of these dopaminergic neurons, we obtained whole-brain images of the brain sample using HD-fMOST imaging (Fig. 3E). We next registered the confocal imaging to HD-fMOST data in order to obtain the coordinates of the recorded dopaminergic neurons. We first selected the images from HD-fMOST data according to the GRIN lens track and projected along the track. Then, we manually registered the confocal images with the HD-fMOST data based on the neuronal distribution and morphology (Fig. 3C). Five of the 7 recorded dopaminergic neurons were registered with HD-fMOST data.

Among the five neurons registered, we successfully reconstructed the entire morphology of 2 dopaminergic neurons (Fig. 3F and S10C). For the other 3 neurons, their axons were partly reconstructed due to weak fluorescence intensity or the poor signal-to-noise ratio around the GRIN lens track (Fig. S10 D-G). In addition, we also fully reconstructed another 2 dopaminergic neurons, which were not imaged *in vivo* but laid below the GRIN lens (Fig. S10H). All of the 4 fully reconstructed dopaminergic neurons had major projections to the striatum, although there were minor differences in their projection target, similar to a previous study [31] (Fig. 3E-G). Furthermore, we observed that the dopaminergic neuron excited by foot-shock has a higher projection probability to the ipsilateral dorsal striatum. Thus, by combining molecular-specific labeling strategy, deep brain imaging, and HD-fMOST, we successfully mapped the brain-wide projectome of SNc neurons with distinct response patterns.

## Discussion

To obtain the brain-wide projectome as well as the neural dynamics for individual neurons is crucial for understanding the working principle of the brain. Here we developed strategies to link brain-wide projectome with neural dynamics for individual neurons, combining viral expression-based labeling, *in vivo* deep brain calcium imaging, and HD-fMOST imaging. The aberration introduced by the GRIN lenses was corrected through AO imaging, facilitating the cell registration between *in vivo* imaging and HD-fMOST. The method developed in our study provided feasible approaches for linking projectome to dynamics in subcortical brain areas, and extended what has been achieved in the previous studies that only focused on the cortical neurons [7, 8].

In our study, we utilized jGCaMP7b as both a functional and structural indicator, known for its higher baseline fluorescence than GCaMP6s and GCaMP6m [8, 17, 33], which made it more suitable for mapping the entire brain-wide projectome. This is evidenced by our data showing that in fMOST imaging, the labeling by jGCaMP7b was comparable to that by EYFP, a widely used fluorescent indicator for reconstructing full neuronal morphology [34–36]. Thus, it is a feasible approach for the purpose of acquiring the neural dynamics and morphological data with the same indicator. With the accumulation of expression of jGCaMP7b, the neuronal state could be unhealthy [17, 33, 37, 38], which would affect the reliability of long-term recordings. Indicators or viral labeling strategies with minimal effects on cell state would further facilitate determining the neuronal function in long-term recordings.

Microscopy integrated with a GRIN lens enabled us to image deeper subcortical brain regions *in vivo* [9–13, 16]. Here, we used GRIN lenses for neural dynamics recording in the deep brain. However, both on-axis and off-axis aberrations of the GRIN lens degrade the resolution and lead to neuronal morphology distortions. In this study, we achieved aberration corrections in a z-stack image by custom-built AO microscopy, and the neuronal morphology distortions across enlarged FOV were improved. Superior volumetric imaging within full FOV was achieved, which provided more accurate morphology information for cell registration between two-photon imaging and fMOST imaging. However, due to the intrinsic nonuniform aberrations induced by the GRIN lens and the limited correction capacity of the current AO microscope [16, 20, 39, 40], it is still not possible to correct the position-dependent aberrations of full FOV during real-time recording. Developing a method providing synchronous aberration corrections over multiple regions in the FOV of GRIN lenses would enable to recording of neuronal dynamics in full FOV without aberrations, allowing more precise analysis of neuronal responses.

Cell registration is crucial for integrating calcium dynamics and projectome data. In our study, we achieved cell registration with a high success rate (70.5% for S1BF; 98.9% for dCA1; 71.4% for SNc) by a modified version of a previous registration method [8]. For registration in dCA1 and SNc, we first selected the FOV of *in vivo* imaging according to the track of the GRIN lens. This approach would increase the accuracy of registration, particularly when the imaging FOV was smaller than the field for labeled neurons, as the case for SNc. We also used the 2D projection of volumetric images for registration [8, 13]. Developing a 3D information-based registration method will further facilitate accurate registration.

To map the projectome of functionally defined neurons within a molecularly defined ensemble, we used a viral strategy relying on binary recombinases in a Cre mouse line in this study. A previous study used gene-specific promoter-driven Cre virus and Cre-dependent indicator to study the morphology and functional dynamics of SST neurons in L2/3 of the visual cortex [8]. Compared to the method using promoter-driven Cre virus, our strategy could be easily used in other types of neurons labeled by specific Cre lines. Given that the fluorescent signal for some neurons was weak, developing viruses that stably express brighter fluorescent proteins in a Cre-dependent manner would greatly facilitate the complete reconstruction of neuronal morphology and enable a more detailed analysis of neural projections

One limitation of this study is the relatively low throughput for deep brain areas. There are two reasons accounting for the low throughput. Firstly, for the reconstruction of neurons, neurons need to be sparsely labeled. The limited FOV of GRIN lenses resulted in only a small portion of neurons being imaged *in vivo*. Second, some neurons could not be fully reconstructed due to the weak fluorescent intensity of axons in distant projection targets and the poor signal-to-noise ratio of the neurites around the GRIN lens track. Third, the implantation of GRIN lens damaged some neurites close to the lens, leading to failed reconstruction of these neurons. Therefore, some of the labeled neurons could not be used for further analysis, reducing the throughput. Three-photon imaging has emerged as a promising technique for recording neuronal dynamics in the intact brain, providing deeper access than two-photon imaging [41, 42]. This approach could potentially address some of the challenges encountered in dCA1. In addition, developing an imaging method with larger penetration depths would facilitate functional imaging in deep brain regions from the intact brain and provide 3D information for cell registration.

In summary, our study developed strategies to obtain the brain-wide projectome of functionally defined neurons in both cortical and subcortical regions by combining *in vivo* imaging and fMOST imaging. We have also explored the projectome of functionally and molecularly defined neurons. This technological advancement paves the way for the comprehensive study of circuit organization by integrating neural dynamics and connectivity information and represents a promising avenue toward a more profound understanding of the fundamental basis of brain functions.

## Supplementary Information

Below is the link to the electronic supplementary material.

Supplementary file1 (PDF 1,769 kb)
