# Supplementary material for: Link Brain-Wide Projectome to Neuronal Dynamics in the Mouse Brain
Source: Neurosci Bull. 2024 May 31;40(11):1621–34. doi: 10.1007/s12264-024-01232-z (PMC11607363; doi:10.1007/s12264-024-01232-z)
Supplement: Supplementary file 1 — Supplementary file1 (PDF 1,769 kb) [file 12264_2024_1232_MOESM1_ESM.pdf]

Supplementary Materials

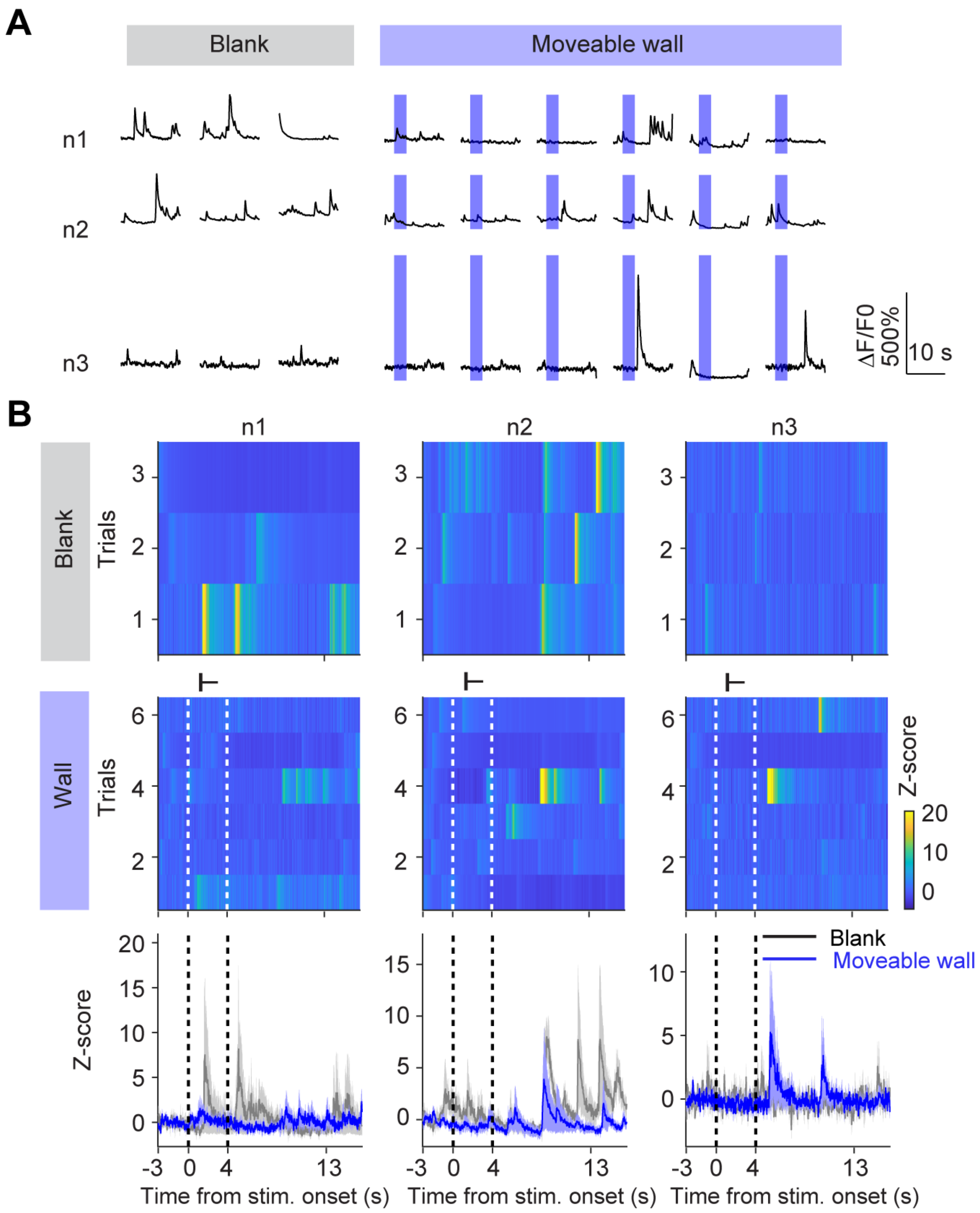

Fig. S1 Calcium dynamics of 3 example neurons in response to moveable wall-delivered touch stimuli in Fig. 1B-D. **A** Neural responses in individual blank or touch stimuli (Moveable wall) trials. **B** Heat maps of the calcium dynamics of neurons in **A** responding to blank controls (Top) or touch stimuli (Movable wall) (Middle). Bottom, Averaged calcium traces of these neurons in blank or touch stimuli trials. Touch stimuli were delivered for 4 seconds as indicated by dashed lines.

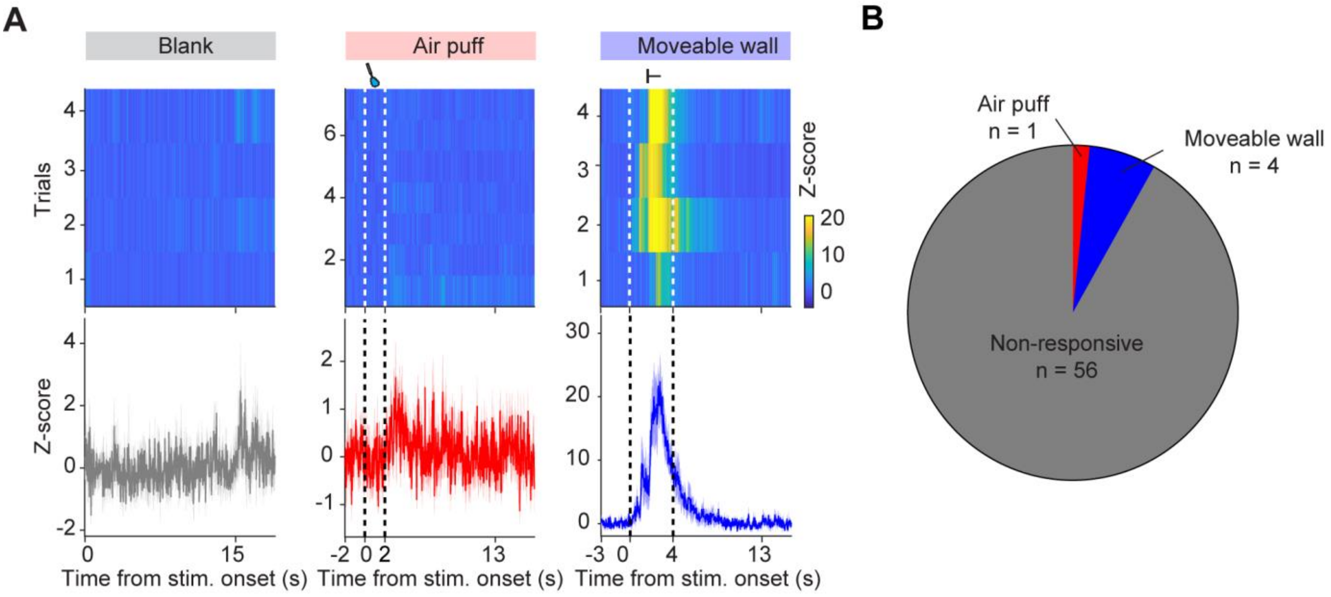

Fig. S2 Neural responses to touch stimuli in L2/3 of S1BF**. A** An example neuron responsive to touch stimuli (Moveable wall). **B** Distribution of recorded neurons in L2/3 of S1BF with distinct functional responsiveness to touch stimuli.

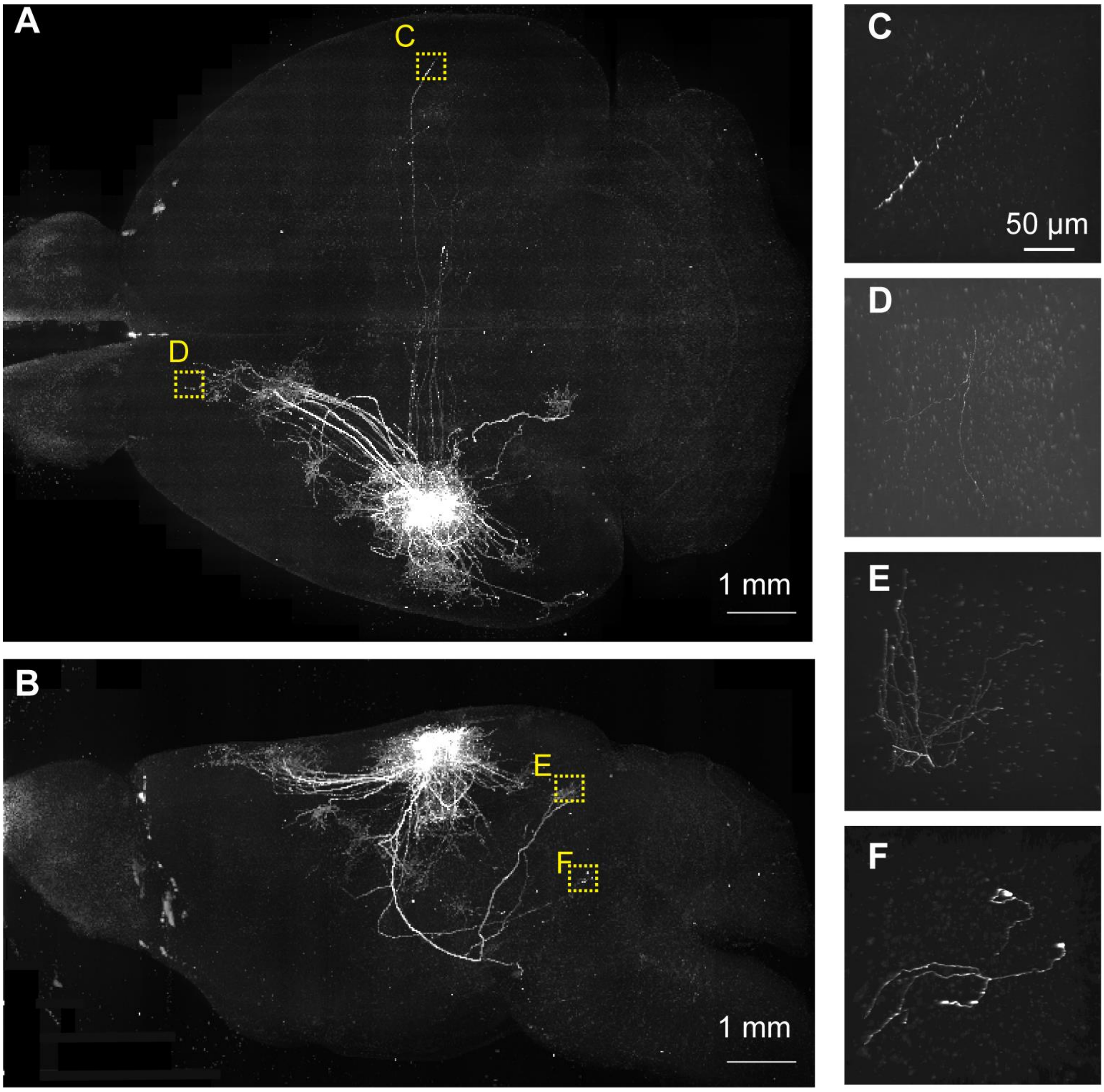

Fig. S3 Raw whole-brain fMOST data of the same sample in **Fig. 1B-D.** **A** and **B** Whole-brain horizontal and sagittal fMOST raw data. **C-F** Enlarged view of terminals indicated in **A** and **B**.

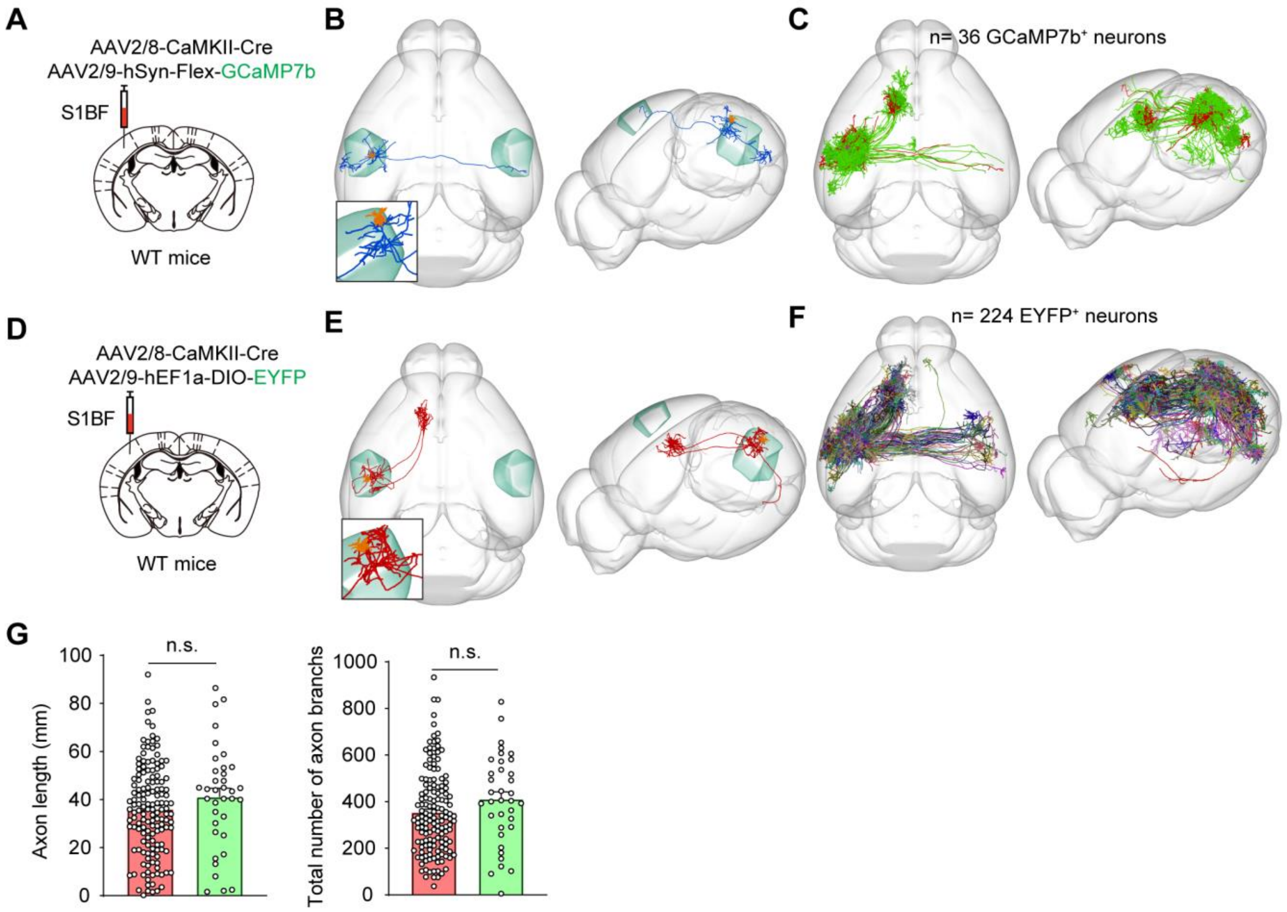

Fig. S4 Morphology of neurons in L2/3 of S1BF indicated by jGCaMP7b or EYFP**.** **A** Schematic diagram of viral mixture (AAV-CaMKII-Cre and AAV-hSyn-Flex-jGCaMP7b) injection in S1BF of wild-type mice. **B** Morphology of an example jGCaMP7b^{+} neurons. The orange line indicates the dendrite. **C** The whole-brain projectome of 36 jGCaMP7b^{+} neurons acquired via fMOST. **D** Schematic diagram of viral mixture (AAV-CaMKII-Cre and AAV-DIO-EYFP) injection in S1BF of wild-type mice. **E** Morphology of an example EYFP^{+} neurons. The orange line indicates the dendrite. **F** The whole-brain projectome of 224 EYFP^{+} neurons acquired via fMOST. **G** Axonal length (Left) and number of axon branches (Right) of neurons labeled by jGCaMP7b or EYFP indicated in **C** or **F**. Data are mean ± SEM. *t*-test was used in **G**. n.s., not significant.

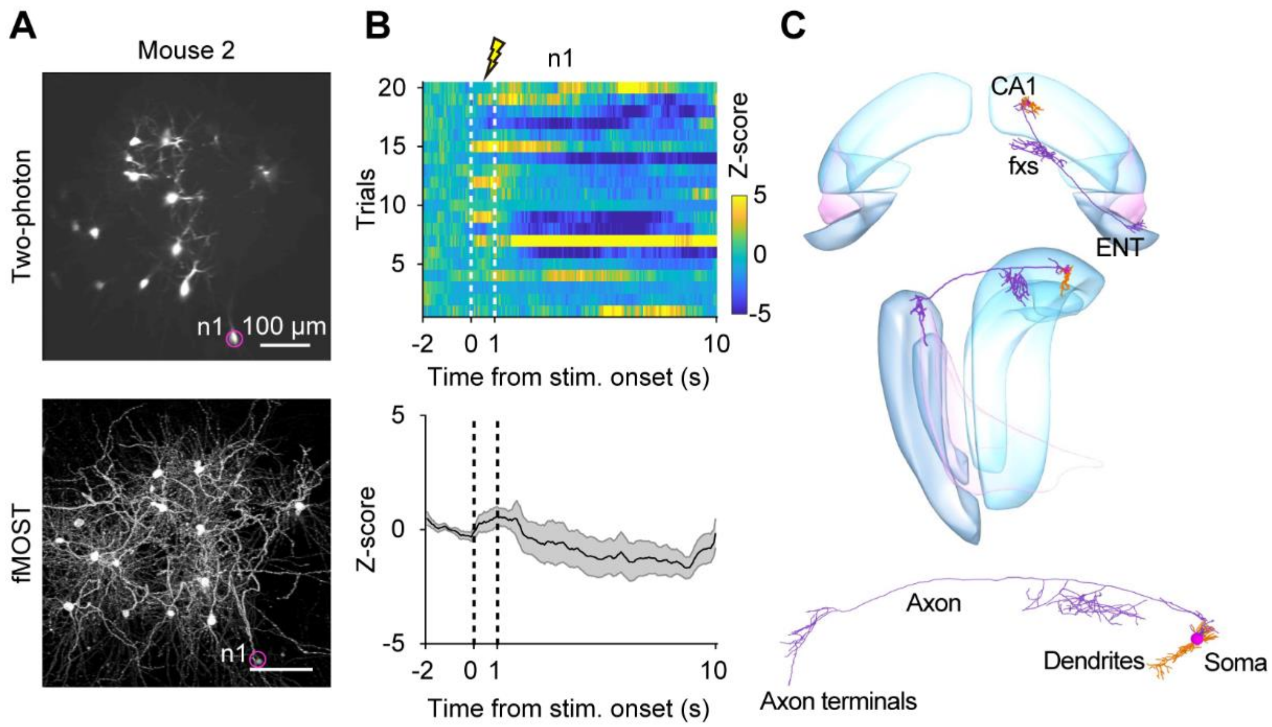

Fig. S5 The representative neuron of NR neuron. **A** Cell registration for neurons between images from two-photon imaging and HD-fMOST of mouse 2. Top, Maximum projection of two-photon z-stack imaging. Bottom, Maximum projection of HD-fMOST imaging around object side of GRIN lens. **B** The heatmap and trace of averaged calcium activity of NR neuron (Mouse 2) indicated in **A** responding to foot-shock. Dashed lines indicate foot-shock delivery. **C** The whole-brain projectome and entire morphology of NR neuron indicated in **B**.

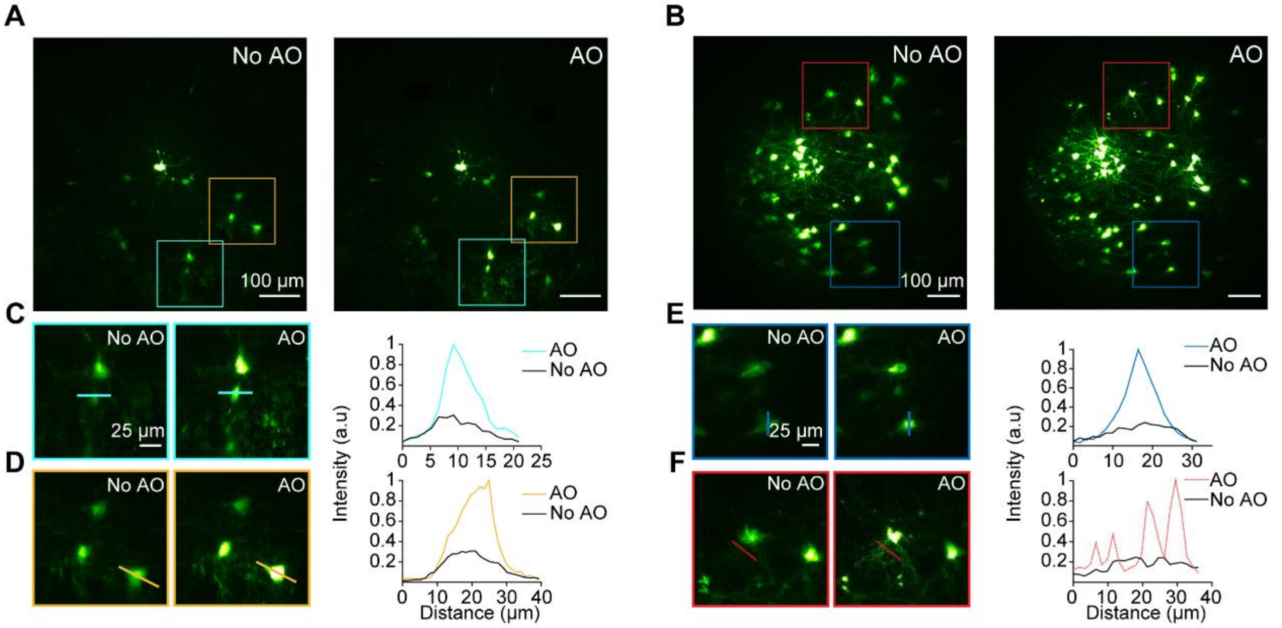

Fig. S6 AO improves *in vivo* two-photon imaging based on GRIN lens. **A** AO correction of sparse neurons located in the center of FOV. **B** AO correction of the neurons located at the edge of FOV. **C** The fluorescence intensity of neurons indicated by cyan lines was increased by approximately 2.29-fold after AO correction. **D** The fluorescence intensity of neurons indicated by orange lines was increased by approximately 2.26-fold AO correction. **E** The fluorescence intensity of neurons indicated by blue lines was increased by approximately 3.15-fold after AO correction. **F** The fluorescence intensity of fibers indicated by the red dash line was increased by approximately 3.06-fold after AO correction.

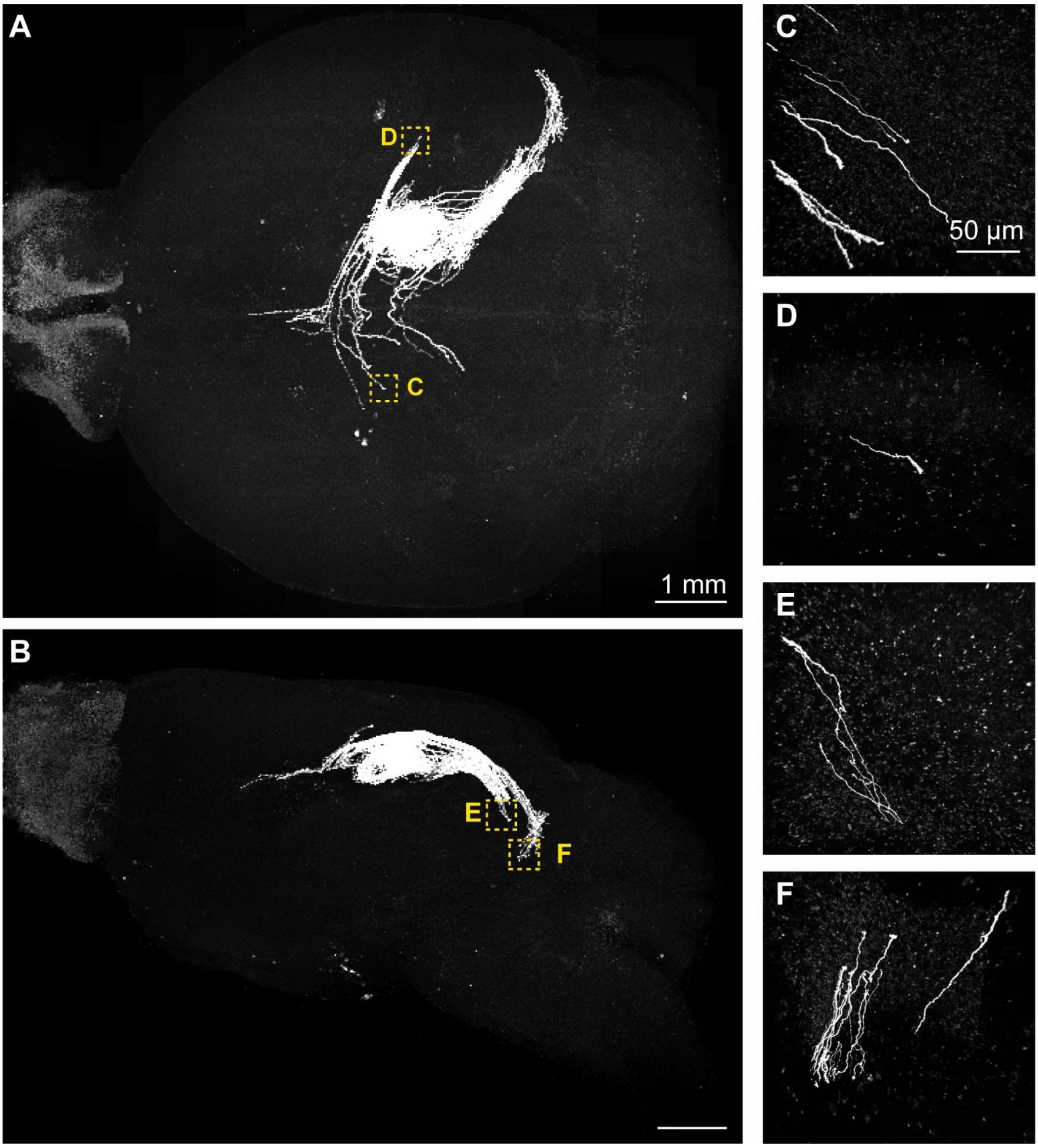

Fig. S7 Raw whole-brain fMOST data of the same sample in Fig. 2B. **A** and **B** Whole-brain horizontal and sagittal fMOST raw data. **C**–**F**, Enlarged view of terminals indicated in **A** and **B**.

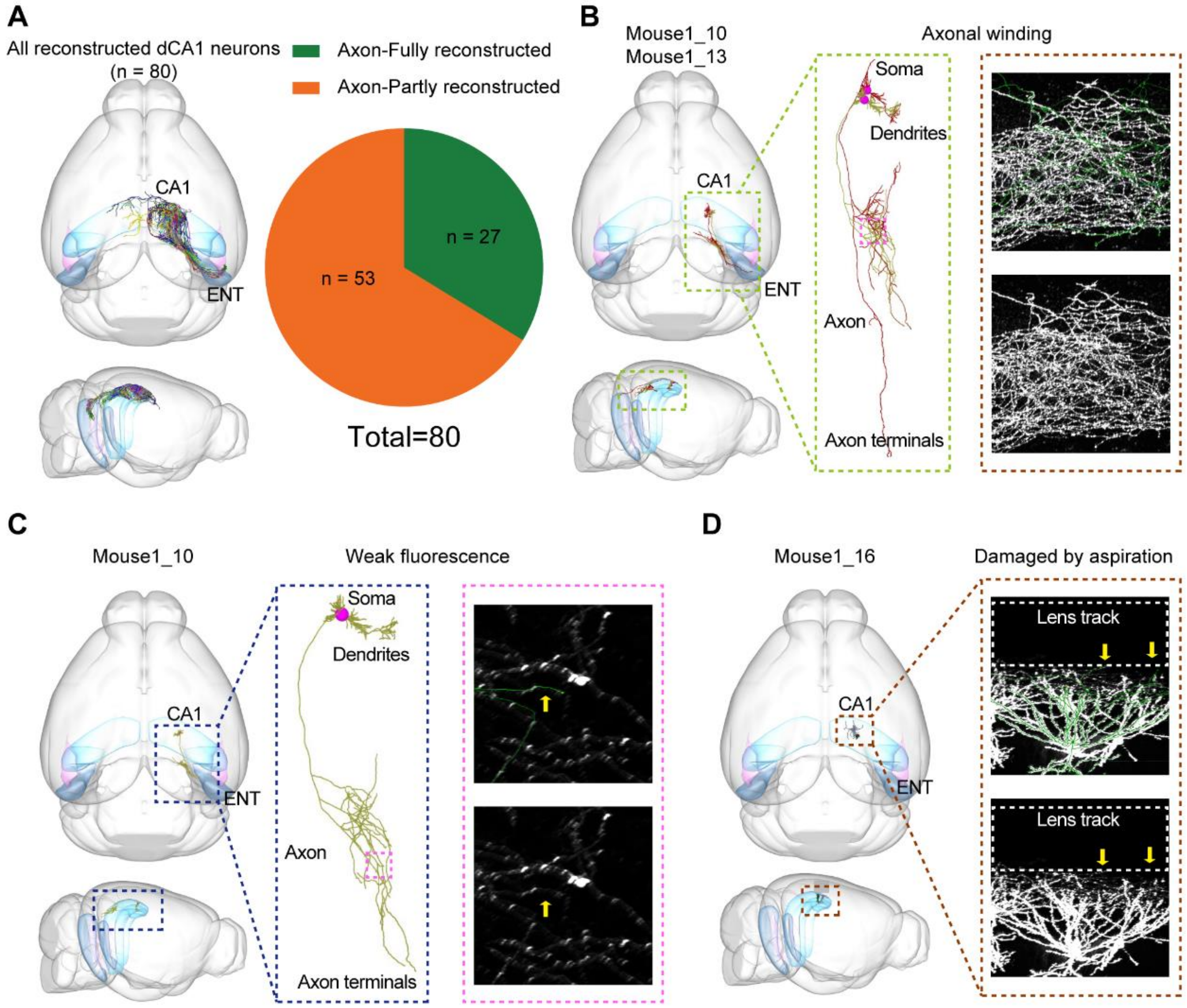

Fig. S8 The projectome of dCA1 neurons. **A** The overview of all reconstructed dCA1 neurons (n = 80). **B** The representative morphology of the dCA1 neuron whose axon could be partly reconstructed due to the axonal winding. **C** The representative morphology of the dCA1 neuron whose axon could be partly reconstructed due to the weak fluorescence. **D** The representative morphology of the dCA1 neuron whose axon could not be reconstructed due to the damage by aspiration of brain tissue above dCA1.

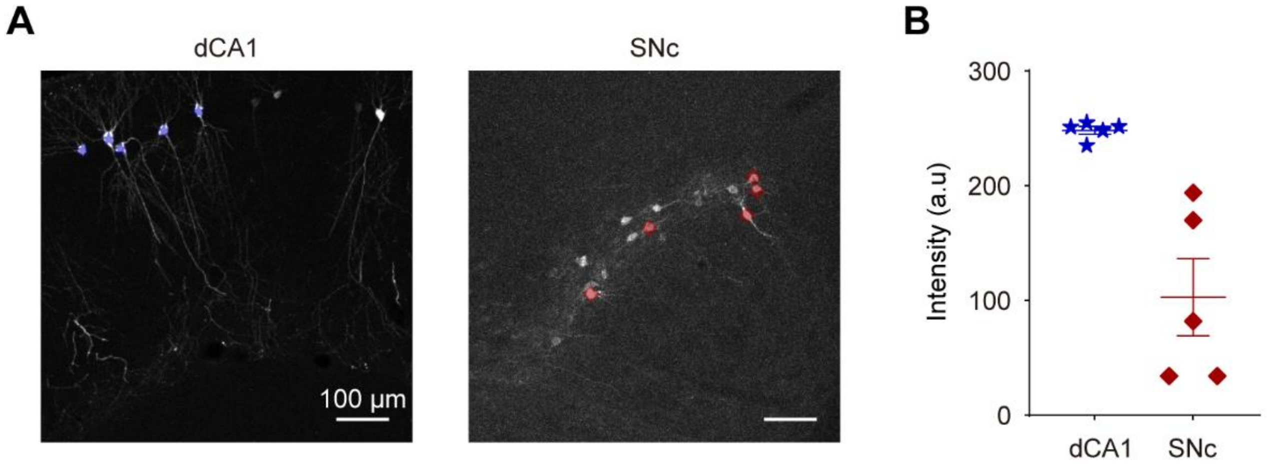

Fig. S9 The neuronal brightness of SNc is weaker than dCA1**. A** The maximum projection of brain slide images in dCA1(Left, power: 8 mW, objective: 10×/0.5 NA) and SNc(Right, power: 20 mW, objective: 20×/0.8 NA). **B** The fluorescence intensity of chosen neurons in dCA1 and SNc is shown in **A**.

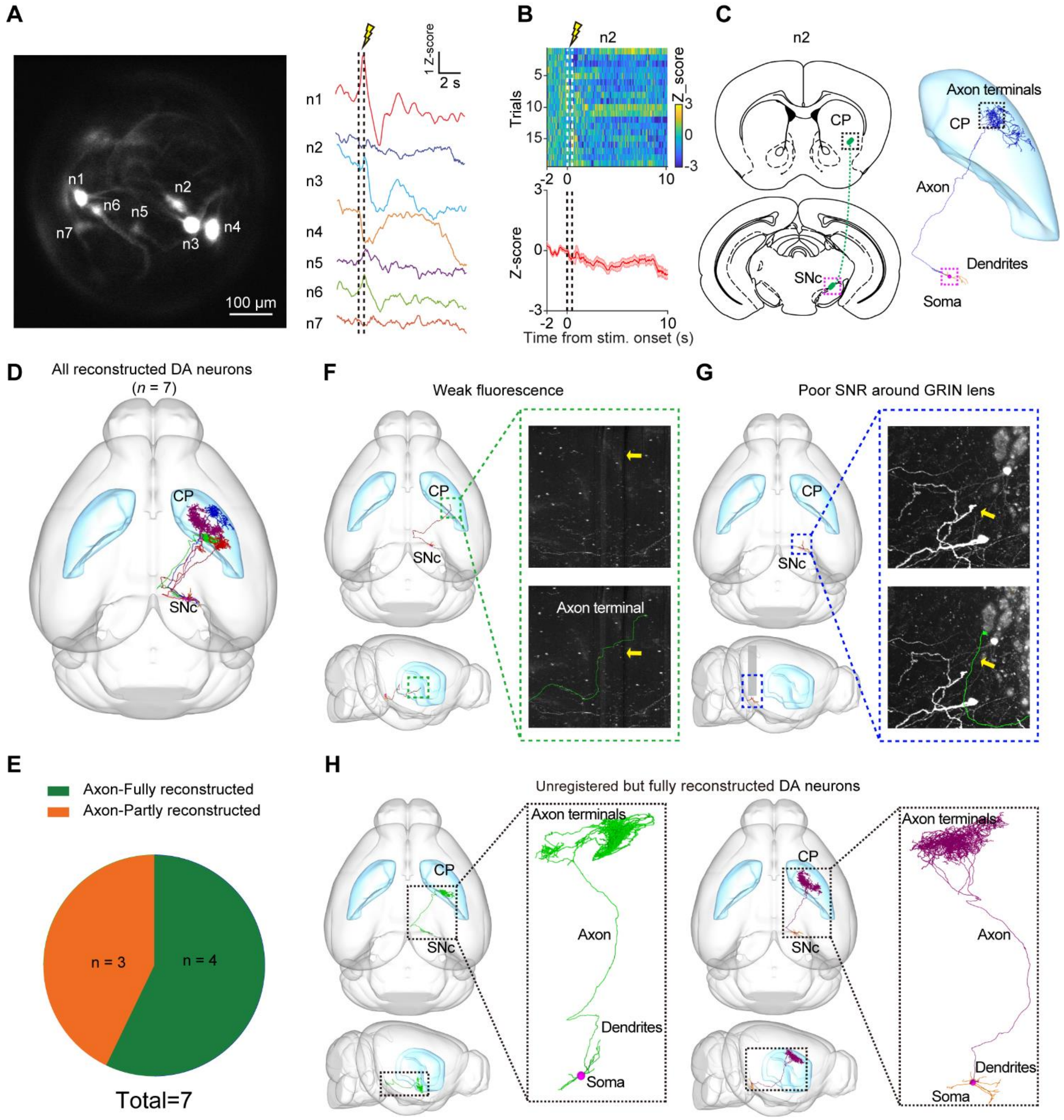

Fig. S10 The dynamic and projectome of SNc dopaminergic neurons. **A** Dynamic responses of SNc dopaminergic neurons to foot-shock stimuli. Left, the average projection of an example imaging session. Right, the averaged trace of recorded neurons for a block of trials (*n* = 19 trials). **B** The heatmap and trace of averaged calcium activity of n2 neuron indicated in Fig.3C responding to foot-shock. Dashed lines indicate foot-shock delivery. **C** The schematic diagram (Left) and whole-brain projectome (Right) of representative dopaminergic neurons corresponding to neurons in **B**. **D** and **E** The overview of all reconstructed dopaminergic neurons (*n* = 8). **F** The representative morphology

of the DA neuron whose axon could be partly reconstructed due to the weak fluorescence of terminals. **G** The representative morphology of the DA neuron whose axon could not be reconstructed due to the poor signal-to-noise ratio around the GRIN lens. **H** The morphology of unregistered DA neurons corresponding to Fig.3G and 3H.
